# Perceptions and attitudes toward clinical trial participation: a study on *Moringa oleifera* Lam. supplementation in adult HIV patients in Kano State, Nigeria

**DOI:** 10.3389/fphar.2025.1676393

**Published:** 2025-10-31

**Authors:** Aisha Gambo, Musa Babashani, Nceba Gqaleni, Limakatso Lebina

**Affiliations:** ^1^ Discipline of Traditional Medicine, School of Medicine, College of Health Sciences, University of KwaZulu-Natal, Durban, South Africa; ^2^ Department of Medicine, Bayero University Kano, Kano, Nigeria; ^3^ Aminu Kano Teaching Hospital, Kano, Nigeria; ^4^ Africa Health Research Institute, Durban, South Africa

**Keywords:** clinical trials, perceptions, participation, PLHIV, Nigeria

## Abstract

**Introduction:**

Participation of patients in well-designed clinical trials forms the essence of all clinical research. PLHIV and receiving ART at AKTH, Kano State, Nigeria, were invited to participate in a six-month *Moringa oleifera* Lam. supplementation study. One hundred and seventy-seven (177) patients participated, while 27 declined. This study assesses the perception and attitude to clinical trial participation as well as the refusal of the study participants.

**Methods:**

A questionnaire interview was conducted on those who participated and those who declined. Data was analysed using both descriptive and inferential statistics.

**Results:**

All the reasons for participation in clinical trials were statistically significant (p < 0.05). Reasons such as “To help myself” and “To improve my health” both have a perfect mean score of 1.0000 and a standard deviation of 0.0000, indicating reasons as the most important for participation. “To help others” (mean = 1.9379) and “Asked by the doctor” (mean = 2.4237) were moderately endorsed. “For the money” (mean = 3.0000) was the least supported among tangible reasons. Most participants understood the consent form, agreed they gained something from participating, felt respected and treated with dignity, trusted that their information would remain private, and believed the research would benefit others. Participation was largely seen as a free and meaningful choice. The most significant reasons cited for the lack of participation were time constraints (p = 0.029) and lack of money to come to the hospital for the study (p = 0.049).

**Discussion:**

Clinical trials can be conducted more quickly and effectively with greater patient participation. Improving awareness will improve patient participation and retention in clinical trials, which will invariably increase access to the newest available treatments.

## 1 Introduction

Participation of patients in clinical trials that are well-designed and well-conducted forms the essence of all clinical research (Unger et al., 2019). Clinical trials are defined as the studies of new tests and treatments and the evaluation of their effects on human health outcomes using approved guidelines (WHO, 2022). It advances medical knowledge and improves patient outcomes by providing a valuable understanding that guides treatment decisions (ICON, 2023). Trials can be conducted more quickly and effectively with greater patient participation, hence enabling the speedy discovery of new treatments. It offers the opportunity for patients to access the latest available treatments (Unger et al., 2019). Furthermore, an umbrella review conducted to determine the benefits of participation in clinical trials suggests that participation may be more beneficial compared to nonparticipation (Bouzalmate-Hajjaj et al., 2022).

Despite the benefits, patient participation, recruitment, and retention in clinical trials remain a challenge. It may result in premature clinical trial termination, unanswered research questions, and wasted limited resources (Briel et al., 2021).

Among the factors that affect the willingness to participate in clinical trials are trust in healthcare providers, which could lead to physician recommendations, family support, and patients with prior clinical trial experience. Studies have reported that lack of awareness, lack of recommendations by physicians, logistical challenges, and a lack of trial accessibility as factors that contribute to the unwillingness of patients to participate (Alotaibi et al., 2025).

Several studies have been reported from different countries on the perception, knowledge, attitudes, and willingness of patients to participate in clinical trials. Al-Shami et al. conducted a cross-sectional study in Jordan to assess the attitude and awareness of the public towards clinical trials. The study also assessed the public’s perceived motivators and barriers to participation in clinical trials. Most of the participants reported being aware of a clinical trial. Top motivators for participation include contributing to science, promoting one’s health, and benefiting others. Time constraints, lack of interest, and fear were the barriers most cited for non-participation (Al-Shami, Ahmed, and Alzoubi, 2022). Similarly, the study reported from Saudi Arabia stated a satisfactory level of knowledge about the benefits of clinical trial amongst participants, and the vital elements of informed consent, with most respondents willing to participate in a clinical trial after consulting their family physician (AL-Tannir et al., 2016). Other studies from Poland reported the opportunity for constant or better care and accessibility to additional monitoring as driving factors for participation, while fear of being used as a “guinea pig” and toxicity of study intervention as main reasons for lack of participation (Kotowski et al., 2019). Feeling of safety and being driven by necessity were reasons for participation as reported by Chu et al. (Chu et al., 2015).

In Africa, literature is lacking on the perception and attitude toward clinical trial participation. Even though Africa carries a significant global disease burden, the lack of literature could be due to a low prevalence of patient participation in clinical trials that is experienced in the region, as only around 4% of international clinical trials are carried out in Africa (World Economic Forum, 2024).

Nigeria has a population of 227.8 million people as of 2023, with a projected population increase of 58% by 2050 (WHO, 2024). This population offers a rich and mostly naïve patient pool with a varied range of disease conditions, which could serve as an advantage for the conduct of clinical trials in Nigeria. This will enable speedy patient recruitment as compared to other countries (Schneider, 2024).

Kano State is in the North-western geopolitical zone and the most populous state in Nigeria (KNSG, 2024). Participating in clinical trials provides a valuable opportunity for people due to limited access to medical treatments and medications (Muhammad, 2024). Nonetheless, social and cultural issues such as religious and language barriers and a general lack of awareness about clinical trials have been reported to impede participation in clinical research (Gimba et al., 2021). Furthermore, available literature from other parts of the world may not be generalizable to these settings, as there are great cultural differences and varying perceptions worldwide, and what is deemed acceptable in one region may not be in another. For instance, certain interventions that have proven effective in high-income nations may not have the same level of success when applied in different settings (Alemayehu et al., 2018). Therefore, strategies to understand the motivation and barriers to clinical trial participation of study participants will assist researchers in the planning and conduct of studies to make better utilization of the patient pool evident in the country.

Previously, we conducted a six-month randomised clinical trial in Kano State to evaluate the effect of *Moringa oleifera* Lam. leaves supplementation on the anthropometric and immune status of adult HIV patients on ART (Gambo et al., 2021). This study is a follow-up aimed at assessing the perception and attitudes to clinical trial participation, or not, of the study participants.

## 2 Methods

A questionnaire interview was conducted at the S.S. Wali Virology Centre at the Aminu Kano Teaching Hospital, Kano State (AKTH), Nigeria, between June and November 2018.

It forms part of a follow-up to the randomised controlled *Moringa oleifera* Lam. supplementation clinical trial. It was approved by the Ethics Committees of Aminu Kano Teaching Hospital (AKTH), Kano, Nigeria (reference number NHREC/21/08/2008/AKTH/EC/2012), and the Biomedical Research Ethics Committee of the University of KwaZulu-Natal, Durban, South Africa (reference number BFC294/16). The clinical trial was registered with the Pan African Clinical Trial Registry (identification number PACTR201811722056449).

The study participants were divided into 2 groups [Group A and Group B]. A detailed description of the participants’ flow using the flow chart was provided in a previous study (Gambo et al., 2021) and the supplementary document in the current study. The flow chart described in detail the procedure used to obtain the participants for the current study. Briefly, Group A comprises those who participated and completed the clinical trial (n = 177). Participants completed a semi-structured questionnaire that was generated from previous studies (Bevan et al., 1993; Tohid et al., 2017) with modifications. The questionnaires were available in English and Hausa and comprised 2 sections. In Section 1, participants were asked to rate the motivations for their clinical trial participation on a scale of 1–3, from most important to third most important. In Section 2, participants were asked to rate a set of 23 pre-defined agreement and disagreement statements relating to their clinical trial experience. Data was collected at the last clinical trial hospital visit of each participant.

Group B comprises those patients who did not participate by either refusing or withdrawing from the clinical trial. It includes eligible participants who refused to participate (n = 6); participants who were lost to follow-up from both study arms (n = 17), and those who discontinued taking the study intervention from both study arms (n = 6). Two (2) participants were unable to be contacted for the interview for the current study; therefore, the total no of patients in group B (n = 27). Participants were asked reasons for declining to participate in the clinical trial with a “YES” or “NO” response. Data was collected telephonically. Patient contact information was retrieved from the patient eligibility, recruitment, and enrollment records.

### 2.1 Statistical analysis

Data entry was done using Microsoft Excel version 2505 and exported for analysis into SPSS version 26.0. Descriptive and Inferential statistics were computed. The demographic characteristics, responses on attitudes and experiences were summarised using frequencies, percentages, means, and standard deviations. Chi test was used to determine the differences between groups, and the p-values were significant at less than 0.05. Mean differences and regression analysis were used to determine the impact of motivation and experiences on attitude. Bar charts were used to visually present mean scores and variations in participant responses.

## 3 Results

Table 1 shows the demographic profile of all respondents who participated in the current study. A total of 204 patients participated in the study. The majority were females (75%) with a predominant age group of 30–39 years (40.2%). In terms of educational level, (62/204) of the participants had a secondary level, and (31/204) were uneducated. 27.5% were traders, 20.6% were artisans, and 19.1% of the study participants were unemployed (Table 1).

**TABLE 1:** Demographic profile of respondents who participated in the moringa clinical trial (Group A, n = 177) and respondents who declined to participate in the moringa clinical trial (Group B, n = 27).

Category		Total participants (N = 204)	Group A	Group B
		Freq (%)	Freq (%)	Freq (%)
Gender	Male	51 (25)	40 (22.6)	11 (40.7)
	Female	153 (75)	137 (77.4)	16 (59.3)
Age	<20years	7 (3.4)	04 (2.3)	03 (11.1)
	20–29years	50 (24.5)	45 (25.4)	05 (18.5)
	30–39years	82 (40.2)	73 (41.2)	09 (33.3)
	40–49years	49 (24)	42 (23.7)	07 (25.9)
	50–60years	16 (7.8)	13 (7.3)	03 (11.1)
Education	Primary	33 (16.2)	26 (14.7)	03 (11.1)
	Secondary	62 (30.4)	51 (28.8)	11 (40.7)
	Tertiary	46 (22.5)	41 (23.2)	05 (18.5)
	Qur’anic	32 (15.7)	28 (15.8)	04 (14.8)
	None	31 (15.2)	31 (17.5)	04 (14.8)
Occupation	Entrepreneur	31 (15.2)	25 (14.1)	06 (22.2)
	Trader	56 (27.5)	48 (27.1)	08 (29.6)
	Civil Servant	36 (17.6)	32 (18.1)	04 (14.8)
	Artisan	42 (20.6)	36 (20.3)	06 (22.2)
	Unemployed	39 (19.1)	36 (20.3)	03 (11.1)
Prev. participation in Clinical Trails	No	-	155 (87.6)	20 (74.1)
	Yes	-	22 (12.4)	07 (25.9)
Disease	KDY	-	22 (12.4)	04 (14.8)
	None	-	155 (87.6)	20 (74.1)
	COTRIM	-		02 (7.4)
	TB	-		01 (3.7)

In those who participated in the *Moringa oleifera* Lam. clinical trial (Group A, n = 177), the mean age of participants was 30–39 years with a female predominance of 77.4%. The level of education was 28.8% and 23.2% having secondary and tertiary level, respectively. Most were traders (48/177) and artisans (36/177). 87.6% had never participated in a clinical trial with only (22/177) who had prior participation in a kidney disease (KDY) clinical trial (Table 1).

In those who declined to participate or withdrew from the *Moringa oleifera* Lam. clinical trial (Group B, n = 27), (16/27) were females with a mean age of 30–39 years. 40.7% of the participants had a secondary level of education. (8/27) were traders and (6/27 and 6/27) were entrepreneurs and artisans respectively. (7/27) had previously participated in kidney (14.8%), tuberculosis (3.7%), and cotrimoxazole (septrin) (7.4%) clinical trials (Table 1).

**FIGURE 1:**
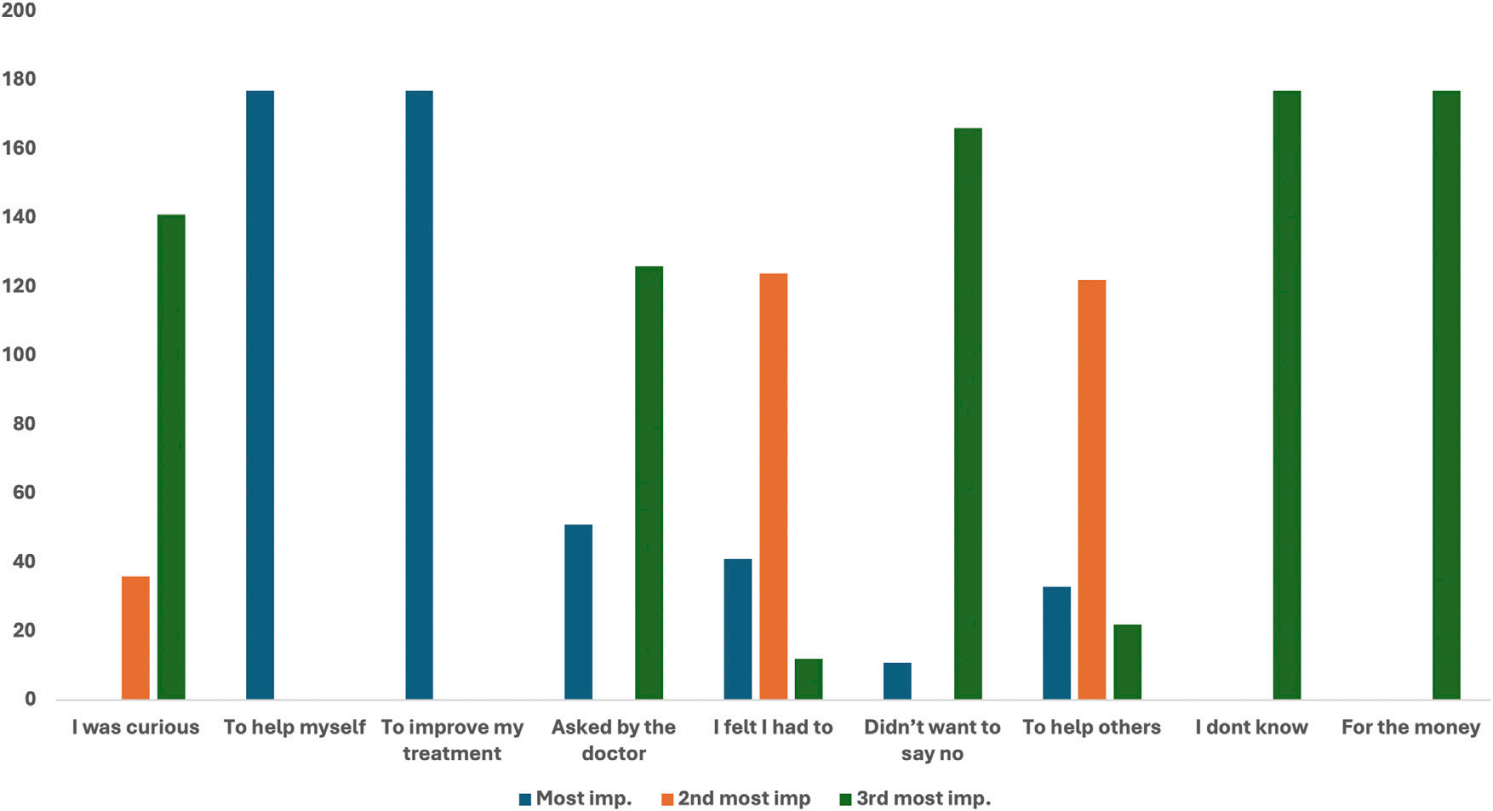
Reasons for participation rated on a“3-point importance ranking scale”.

Figure 1 shows the reasons for participation in clinical Trials. Group A was requested to rate their reasons for participation on a “3-point importance ranking scale,” where 1 was “the most important,” 2 was “second most important,” and 3 was “third most important,” factor. All 177 (100%) participants who were involved in the trials revealed that their reasons for participation were “to help myself” and “I thought it might improve my treatment.” The other reasons attributed to participation were” “I felt I had to” (124/177) and “to help others” (122/177). All 177 (100%) participants revealed that “For the money” was not a reason for their participation in the clinical trial. “I did not want to say no” (166/177) and “I was curious” (141/177) were also the least important reasons for their participation (Figure 1).

**FIGURE 2:**
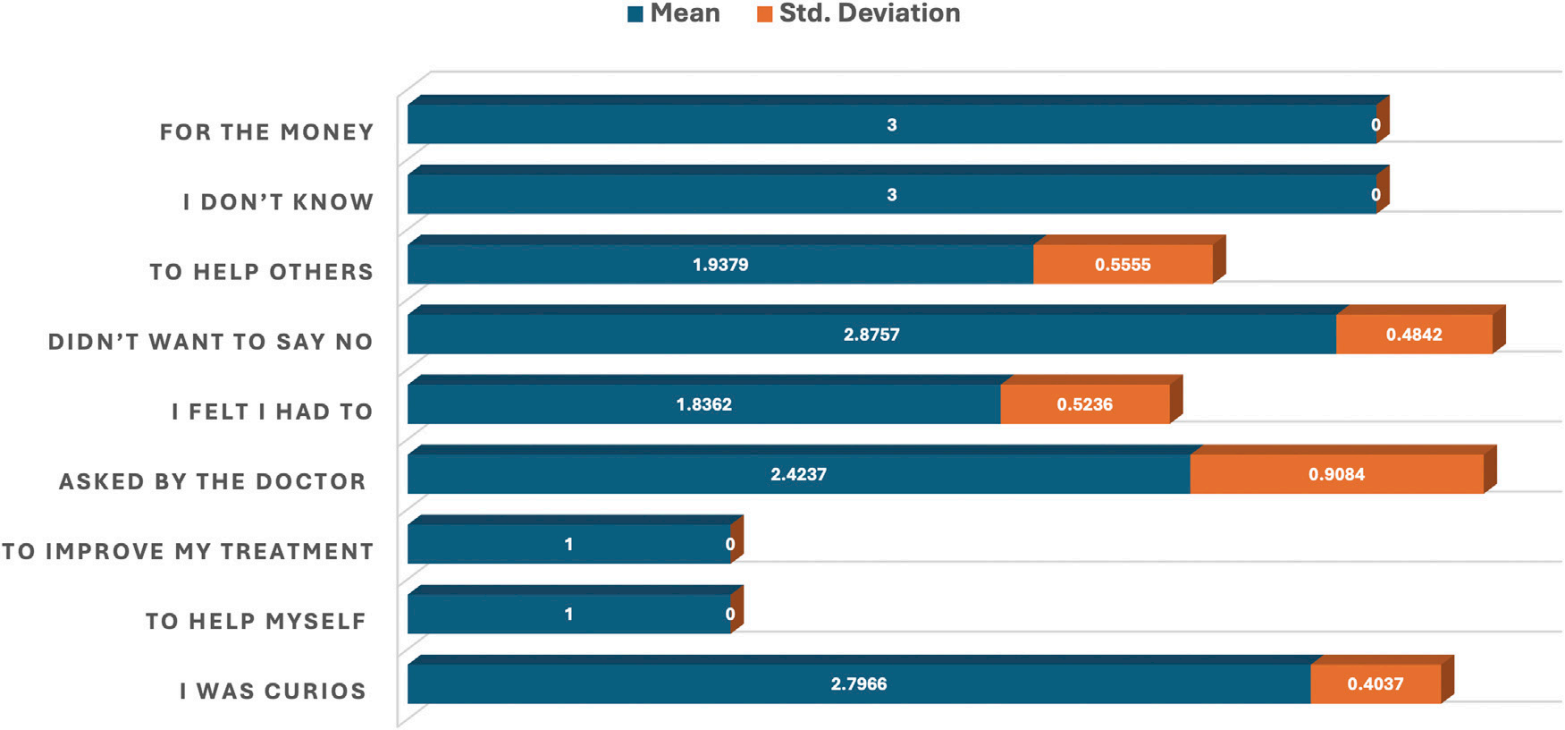
Bar plot of the mean and standard deviation showing reasons for participation in clinical research.

Table 2; Figure 2 provide reasons why Group A participants chose to participate in clinical research, as assessed through mean responses, standard deviations, and p-values. All items listed have statistically significant p-values (p < 0.05). Reasons such as “To help myself” and “To improve my health” both have a perfect mean score of 1.0000 and a standard deviation of 0.0000, indicating that these reasons are most important for participation. Reasons which include “To help others” (mean = 1.9379), “I felt I had to” (mean = 1.8362), and “Asked by the doctor” (mean = 2.4237) were moderately endorsed.

**TABLE 2:** The different reasons for participation in clinical research.

Reasons	Mean ± Std. Deviation	P-value
To help myself	1.0000 ± 0.0000	0.000
To improve my text	1.0000 ± 0.0000	0.000
I felt I had to	1.8362 ± 0.5236	0.000
To help others	1.9379 ± 0.5555	0.000
Asked by the doctor	2.4237 ± 0.9084	0.000
I was curious	2.7966 ± 0.4037	0.000
Didn’t want to say no	2.8757 ± 0.4842	0.000
I don’t know	3.0000 ± 0.0000	0.000
For the money	3.0000 ± 0.0000	0.001

Least important reasons but still statistically significant were responses like “I was curious” (mean = 2.7966) and “Didn’t want to say no” (mean = 2.8757). “For the money” (mean = 3.0000) was the least supported among tangible reasons (Table 2; Figure 2).

Table 3 reveals the relationship between various demographic profiles and respondents’ agreement or disagreement with statements related to participation in clinical research. Most of the demographic characteristics, including gender (p = 0.209), age (p = 0.976), education (p = 0.804), and occupation (p = 0.332), were not significantly associated with respondents’ views. However, the variable previous participation in clinical trials had a statistically significant (p = 0.002) association with the agreement or disagreement statements (Table 3).

**TABLE 3:** Comparison of demographic profile of surveyed population on the agreement and disagreement statements related to participation in clinical research.

Category		Freq (%)	P-value
Gender	Male	40 (22.6)	0.209
	Female	137 (77.4)	
Age	<20years	04 (2.3)	0.976
	20–29years	45 (25.4)	
	30–39years	73 (41.2)	
	40–49years	42 (23.7)	
	50–60years	13 (7.3)	
Education	Primary	26 (14.7)	0.804
	Secondary	51 (28.8)	
	Tertiary	41 (23.2)	
	Qur’anic	28 (15.8)	
	None	31 (17.5)	
Occupation	Entrepreneur	25 (14.1)	0.332
	Trader	48 (27.1)	
	Civil Servant	32 (18.1)	
	Artisan	36 (20.3)	
	Unemployed	36 (20.3)	
Prev. participation in Clinical Trails	No	155 (87.6)	0.002
	Yes	22 (12.4)	

**FIGURE 3:**
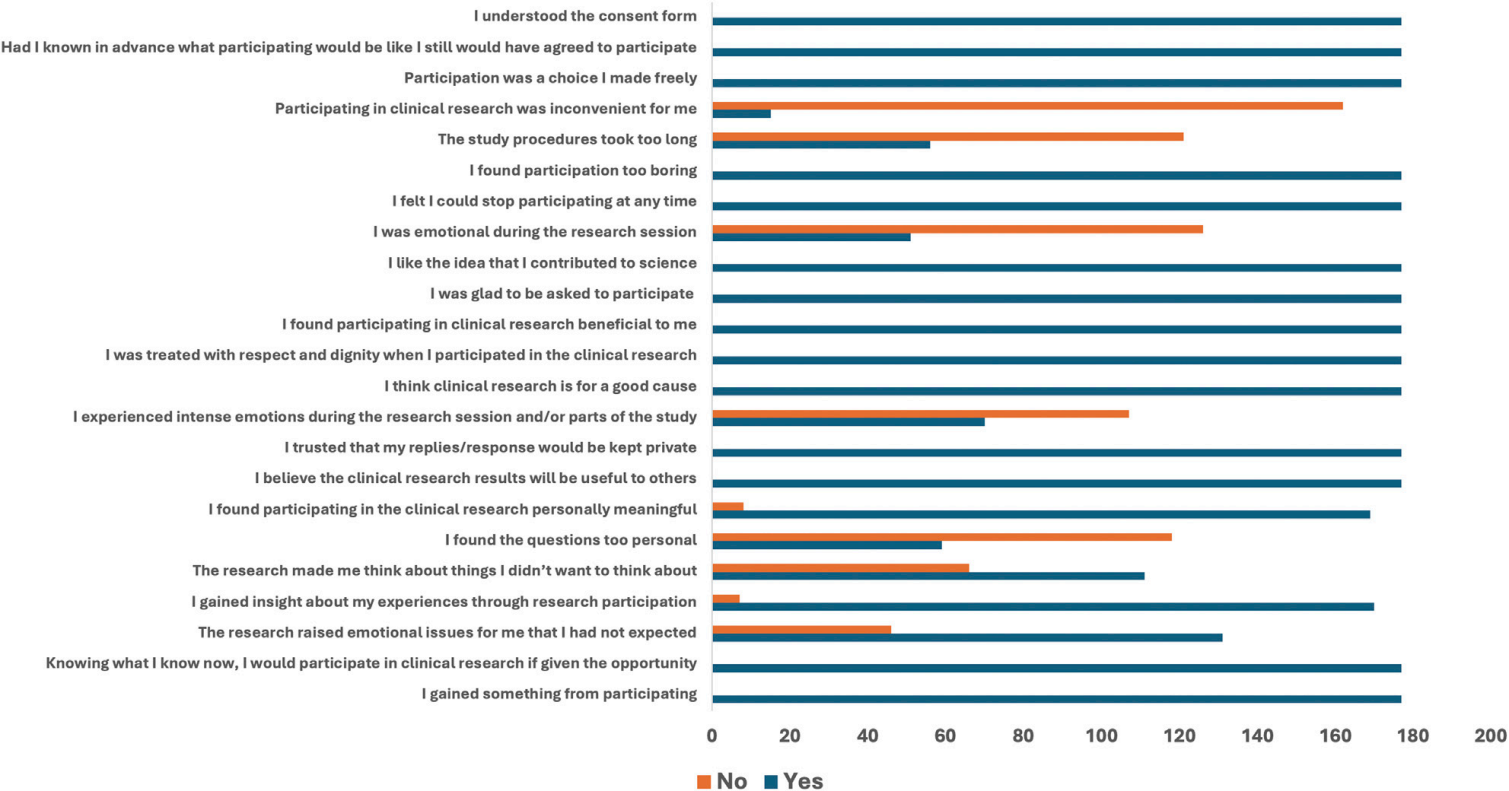
Frequency distribution of agreement and disagreement statements to participation in a clinical trial.

Figure 3 shows the frequency distribution of participants’ responses to the various agreement and disagreement statements related to participation in clinical trials using a bar chart.

All 177 (100%) participants who participated in the trials responded with a “YES” to statements that included “I gained something from participating,” “Knowing what I know now, I would participate in clinical research if given the opportunity,” “I believe the clinical research results will be useful to others,” “I trusted that my replies would be kept private,” “I think clinical research is for a good cause,” “I was treated with respect and dignity,” “I found participating in clinical research beneficial,” “I was glad to be asked to participate,” “I like the idea that I contributed to science,” “I felt I could stop participating at any time,” “Participation was a choice I made freely,” “Had I known in advance … I still would have agreed,” and “I understood the consent form.”

On the other hand, participants disagreed with some statements with a “NO” which include: “I found the questions too personal” (118/177), “I experienced intense emotions during the research session” (107/177), “I was emotional during the research session” (126/177), “The study procedure was too long” (121/177), and “Participating in clinical research was inconvenient for me” (162/177) (Figure 3).

**FIGURE 4:**
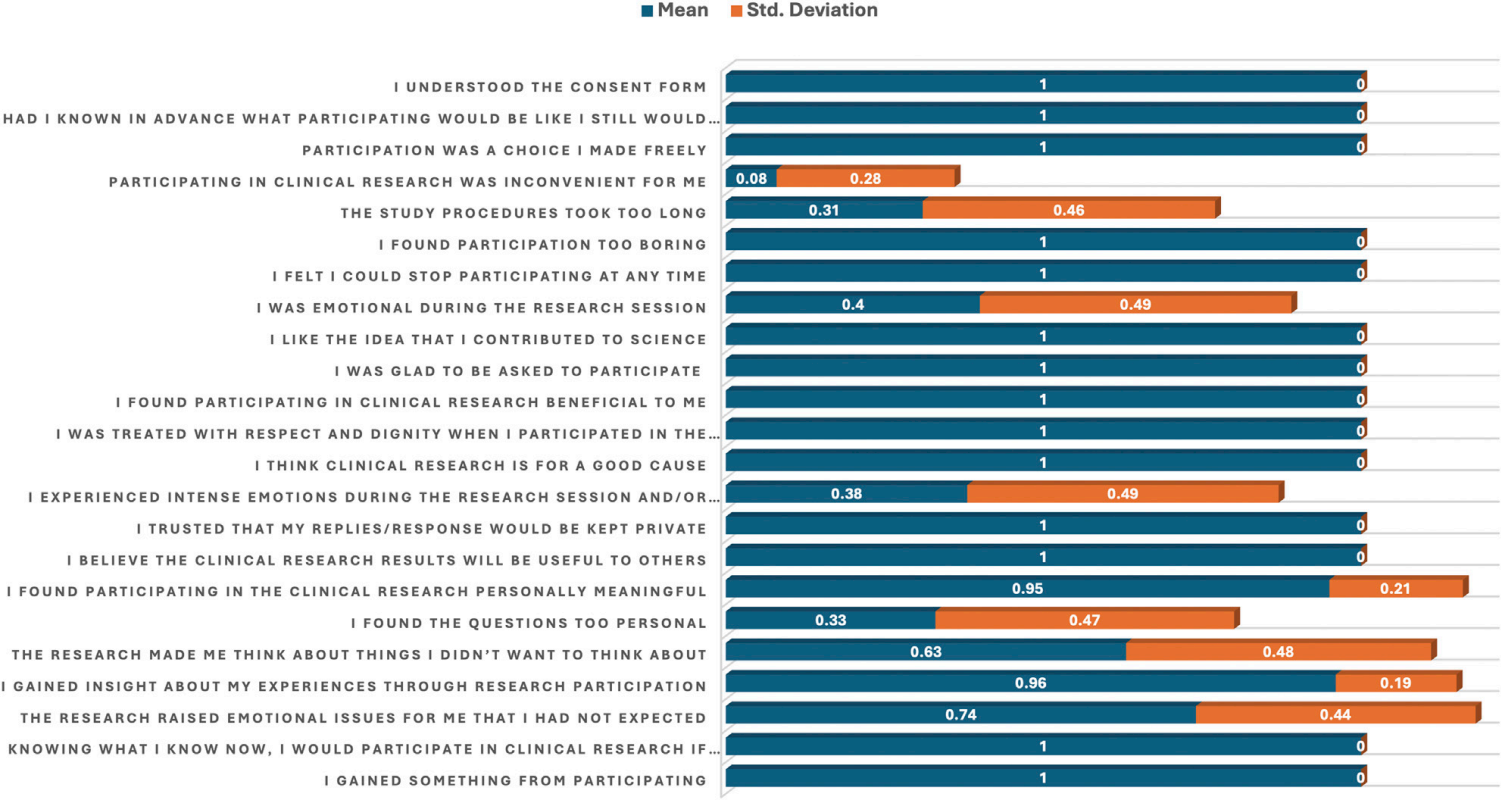
Bar plot of the mean and standard deviation showing agreement and disagreement statements related to participation in clinical research.

Table 4; Figure 4 provide participants’ responses to agreement or disagreement statements regarding their involvement in clinical research using descriptive statistics (means and standard deviations) and p-values to evaluate the strength and relevance of each statement.

**TABLE 4:** Agreement and disagreement statements related to participation in clinical research.

Statements	Mean ± Std. Deviation	P-value
I gained something from participating	1.00 ± 0.00	1.000
I believe the clinical research results will be useful to others	1.00 ± 0.00	1.000
I trusted that my replies/response would be kept private	1.00 ± 0.00	1.000
I think clinical research is for a good cause	1.00 ± 0.00	1.000
I was treated with respect and dignity when I participated in the clinical research	1.00 ± 0.00	1.000
I found participating in clinical research beneficial to me	1.00 ± 0.00	1.000
I was glad to be asked to participate	1.00 ± 0.00	1.000
I like the idea that I contributed to science	1.00 ± 0.00	1.000
I found participation too boring	1.00 ± 0.00	1.000
Participation was a choice I made freely	1.00 ± 0.00	1.000
Had I known in advance what participating would be like I still would have agreed to participate	1.00 ± 0.00	1.000
I understood the consent form	1.00 ± 0.00	1.000
Knowing what I know now, I would participate in clinical research if given the opportunity	1.00 ± 0.00	1.000
I felt I could stop participating at any time	1.00 ± 0.00	1.000
I gained insight about my experiences through research participation	0.96 ± 0.19	0.137
I found participating in the clinical research personally meaningful	0.95 ± 0.21	0.089
The research raised emotional issues for me that I had not expected	0.74 ± 0.44	0.000
The research made me think about things I didn’t want to think about	0.63 ± 0.48	0.000
I was emotional during the research session	0.40 ± 0.49	0.000
I experienced intense emotions during the research session and/or parts of the study	0.38 ± 0.49	0.000
I found the questions too personal	0.33 ± 0.47	0.000
The study procedures took too long	0.31 ± 0.46	0.000
Participating in clinical research was inconvenient for me	0.08 ± 0.28	0.000

Several statements received a perfect agreement score (mean = 1.00, SD = 0.00). These statements include: “I gained something from participating,” “I believe the clinical research results will be useful to others,” “I trusted that my replies would be kept private,” “I think clinical trial is for a good cause” “I was treated with respect and dignity,” “I found participating in clinical research beneficial,” “I was glad to be asked to participate,” “I like the idea that I contributed to science,” “Participation was a choice I made freely,” “Had I known in advance … I still would have agreed,” and “I understood the consent form.” These responses are statistically non-significant (p = > 0.05). However, some statistically significant (p = < 0.05) statements that received agreement scores also include “The research raised emotional issues I had not expected’ (mean = 0.74, p = 0.000),” The research made me think about things I didn’t want to think about’ (mean = 0.63, p = 0.000).

On the other hand, statistically significant (p = < 0.05) statements that received disagreement scores with a “NO” include “I experienced intense emotions during the research session” (mean = 0.38, p = 0.000), and “I was emotional during the research session” (mean = 0.40, p = 0.000). Other significantly endorsed disagreement statements included “I found the questions too personal” (mean = 0.33, p = 0.000), “The study procedures took too long” (mean = 0.31, p = 0.000), and “Participating in clinical research was inconvenient for me” (mean = 0.08, p = 0.000) (Table 4; Figure 4).

Reasons for non-participation or withdrawals from trials (Group B).

In Group B, all 27 (100%) of participants who refused or withdrew from participation in the clinical trial reported that “I am too ill” and “Family objected” were not reasons for their non-participation. Similarly, “I am too old” (26/27), “I don’t like hospitals” (25/27), “I don’t want to change treatment” (23/27), and “fear” (23/27) were not reasons for refusal or withdrawal from participation. Reasons for non-participation were the “lack of enough time” (15/27), “Lack of awareness about clinical research” (12/27), “lack of money to come to the hospital for the research” (9/27), and “possible side effects of tests/drugs” (8/27).

**FIGURE 5:**
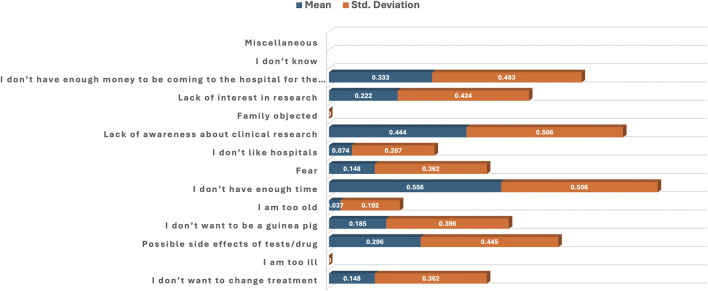
Bar plot of the mean and standard deviation showing reasons for refusal to participate in the clinical trial.

Table 5; Figure 5 present the statistical interpretation of various reasons for non-participation in the clinical trial. Statistically significant disagreement reasons for non-participation were “I am too ill” (mean = 0.000, p = 0.001), “Family objected” (mean = 0.000, p = 0.001), “I am too old” (mean = 0.037, p = 0.004) and “I don’t like hospitals” (mean = 0.074, p = 0.011). Statistically significant reasons for refusal or withdrawal from participation were “I don’t have enough time” (mean = 0.556, p = 0.029) and “I don’t have enough money to be coming to the hospital for the research” (mean = 0.333, p = 0.049). “Lack of awareness about clinical research” (mean = 0.444, p = 0.274) was not statistically significant, but its higher mean value indicates an agreement response to the statement. Other non-significant reasons include “Possible side effects of tests/drugs” (mean = 0.296, p = 0.715), “Lack of research interest” (mean = 0.222, p = 0.274), “I don’t want to change treatment” (mean = 0.148, p = 0.069) and “Fear” (mean = 0.148, p = 0.069) (Table 5; Figure 5).

**TABLE 5:** Reasons for refusal to participate in the clinical trial.

Reasons	Mean ± Std. Deviation	P-value
I don’t have enough time	0.556 ± 0.506	0.029
Lack of awareness about clinical research	0.444 ± 0.506	0.274
I don’t have enough money to be coming to the hospital for the research	0.333 ± 0.483	0.049
Possible side effects of tests/drug	0.296 ± 0.445	0.715
Lack of interest in research	0.222 ± 0.424	0.274
I don’t want to be a guinea pig	0.185 ± 0.396	0.145
I don’t want to change treatment	0.148 ± 0.362	0.069
Fear	0.148 ± 0.362	0.069
I don’t like hospitals	0.074 ± 0.267	0.011
I am too old	0.037 ± 0.192	0.004
I am too ill	0.000 ± 0.000	0.001
Family objected	0.000 ± 0.000	0.001
I don’t know	-	-
Miscellaneous	-	-

## 4 Discussion

Clinical trials on People Living with HIV (PLHIV) are one of the most conducted types of research in Nigeria, yet few studies have examined the perception and attitudes of participants on their clinical trial participation.

Our results reveal that females aged 30–39 years were the dominant participants in the current study. The majority had a secondary school level of education, with a large proportion that were uneducated and mostly traders and artisans.

In those who participated in the *Moringa oleifera* Lam. clinical trial (Group A), the majority were middle-aged, female, educated, and mostly traders and artisans. Almost all the participants had no previous clinical trial participation experience, with a few that participated in a kidney disease clinical trial. The gender distribution reported in our study is not in conformity with studies that reported a predominantly male (AL-Tannir et al., 2016), or approximately equal gender distribution amongst study participants (Tohid et al., 2017; Al-Shami et al., 2022). These studies were conducted in the Middle East, where certain patriarchal cultures could be responsible for the dominant male representations in clinical trials. Additionally, the varied occupational background and educational background, indicating a substantial number of participants with Quranic knowledge and even those without any formal education, reported in our study highlights the importance of inclusive communication strategies during trial recruitment. Quranic education which is acquired by a significant Muslim participants reflect study diversity. Trial recruitment should aim to widen the talent pool by recruiting candidates from diverse backgrounds to give everyone an equal opportunity to participate (Scott, 2025).

Notably, a large proportion of participants with no prior clinical trial participation (87.6%) volunteered to take part in the current study. This demonstrates a willingness among the general population to engage in research when adequately informed. Our results agree with a study conducted in Jordan, where a small number of participants (21%) had previously participated in a clinical trial (Al-Shami et al., 2022). DasMahapatra et al. reported a willingness to participate in clinical trials of people with no prior trial experience, as very few are invited to participate. The study reported that patients with chronic illnesses expressed interest in learning more about participating in clinical trials and indicated a willingness to take part in a trial in the next 12 months (DasMahapatra et al., 2017).

Participants in Group B, representing those who refused or withdrew from participating in the clinical trial, demonstrated a similar demographic characteristic to that of Group A. Only a few reported having previously participated (4%) in a variety of clinical trials, including Kidney, cotrimoxazole, and Tuberculosis. It was reported that previous bad experiences relating to mistrust in the method of obtaining informed consent or attaining a trusting relationship with a healthcare provider could be reasons for declining to participate in clinical trials (Chu et al., 2015). Also, unreported side effects of the study intervention could be reasons for withdrawing from the study.

We observed that none of the examined demographic variables exhibited statistically significant relationships with the various reasons for participation in the clinical trial. This suggests that respondents’ motivations to participate were not significantly influenced by their demographic profile, implying that other factors beyond gender, age, education, occupation, or prior experience may better explain their decision to engage in the study. Gouveia et al. reported a difference in the psychological characteristics of patients who are willing to participate in clinical trials from those who refused to participate. Patients who participate are specifically more self-efficacious, curious, less anxious, and have a higher level of social support (Gouveia et al., 2022).

The various reasons why individuals chose to participate in clinical trials, as assessed, were all statistically significant. This indicates that each reason had a meaningful influence on participants’ decisions to engage in clinical trials. Participants strongly agreed that their reasons for participating were to help themselves and to improve their health, suggesting that personal health benefits were a central motivation for participation. Similarly, altruistic motivations like “To help others” and “I felt I had to” indicate a moral or social sense of obligation as an important driver for participation. Similar reasons were reported by Neergaard et al. in a study conducted on people who are accessing Antiretroviral Therapy (ART). The study is an Analytical Treatment Interruption (ATI) type of research, defined as a closely monitored clinical pause in ART, which is a core component of many HIV cure-directed studies. The study poses significant risks both physically and psychosocially to participants. Despite the risks, finding a cure for HIV and helping others in the HIV community were the significant motivating factors for participation in the clinical trial (Neergaard et al., 2023). Additionally, the importance of getting their medical bills covered and receiving feedback on trial results upon completion of the trial were further reasons cited for participation in clinical trials (DasMahapatra et al., 2017).

In accordance with the literature, the *Moringa oleifera* Lam. supplementation clinical trial was generally well received by the population at the study site, mostly due to their knowledge of the perceived health potential of *Moringa oleifera* Lam. Therefore, self-benefit was a strong motivation for participation. Additionally, those who participated in the *Moringa oleifera* Lam. clinical trial have gained increased routine medical care compared to what was usually offered for PLHIV who accessed their ART at the study site. Participants’ CD4 cell counts were measured monthly after each hospital visit, and viral load was measured twice, at the beginning and the end of the study. Literature reported on the possible benefits gained by participation in clinical trials, which include close clinical assessment that may affect the clinical management of any ailment affecting the participant (Schwarz et al., 2021).

Interestingly, monetary benefit was the least supported among tangible reasons, indicating that financial incentives were not a primary factor in the decision to participate. Although our study was conducted in a low-income setting of Kano State, Nigeria, with participants of low socioeconomic status, financial incentive was the least motivating factor for participation. Even though it was indicated in the consent forms that the participants were informed that a stipend would be given to assist in transportation to and from the study site, a few participants displayed surprise when they were offered their stipend during the first two clinic visits. This confirms that other factors, not monetary, drove their decision to participate in the study. Our findings conformed with the studies conducted in Jordan and Qatar, where it was reported that study participants usually turn down reimbursements offered to them. The study stated that monetary benefits were the least important reason for participation as considered by both the Qatari and other Arab nationalities (Tohid et al., 2017; Al-Shami et al., 2022).

The various demographic profiles that were not significantly associated with respondents’ views on agreement or disagreement statements related to participation in clinical research indicate that these factors did not influence how individuals responded to statements about participating in clinical trials. However, previous participation in clinical trials significantly influenced respondents’ positions. These suggest that individuals who had prior experience with clinical research were more likely to hold stronger opinions or perspectives toward future participation. These findings suggest that health-related factors may be more critical than socio-demographic ones in determining participants’ disposition toward the research involvement.

In our study, most participants agreed that they gained something from participating, felt respected and treated with dignity, trusted that their information would remain private, and believed the research would benefit others. Participation was largely seen as a free and meaningful choice, with individuals expressing satisfaction at contributing to science. Participants confirmed that they had understood the consent form. Although these statements were statistically non-significant, their unanimous endorsement reflects very positive participant perceptions of ethics, respect, and benefit in the research process. Additionally, participants claimed the questions were not too personal. Neither were the research procedures too long nor inconvenient. On the other hand, A smaller proportion found certain aspects emotionally challenging as it stirs up unpleasant thoughts. Similar agreeable statements were reported by Tohid et al., but their participants did not state any emotionally challenging experiences (Tohid et al., 2017).

As indicated, the *Moringa oleifera* Lam. study was conducted on PLHIV. While collecting data, some questions that were asked raised emotionally charged psychological outcomes in a few participants. These emotions were mostly in aspects regarding partner mistrust, HIV infection acquisition, and having to live with the fact that the patient must take ART every day for the rest of his/her life.

Even though HIV has become a manageable chronic disease, PLHIV have experienced several challenges that affect their quality of life. Mental health disorders, stigma and discrimination, emotional challenges of daily dosing of ART, and disruptive changes in lifestyle because of HIV treatment are some of the challenges encountered by PLHIV (Rios et al., 2020). Tomoro et al. reported that PLHIV also expresses ‘involuntary privacy disclosure’ as an emotional burden associated with HIV (Toromo et al., 2022).

Literature has reported on various reasons why participants decline to participate in clinical trials. Our results show that being too ill, family objection, and the perception of being “too old” were not reasons for the refusal to participate. This is true because our participants were not bedridden patients. As inclusion criteria for the *Moringa oleifera* Lam. study, patients had to be on ART for more than 3 months with the absence of any active opportunistic infections. Also, the age range of participants was between 20 to 49 years.

The most significant reasons cited for the lack of participation were time constraints and a lack of money to come to the hospital for the study. Some prospective study participants highlighted the difficulty they encountered in requesting time off from their workplaces to visit the hospital. This is more pronounced due to issues of confidentiality regarding their HIV status. They felt that asking for time off frequently may arouse suspicion about their health. Some reported to be coming from a long distance, hence their challenge to participate in the study.

In terms of financial inability, this suggests that these participants did not understand the information on the consent form, as it was clearly indicated that a stipend would be given to assist participants with transportation to and from the study site after each clinic visit. This shows that the clinical research team should invest more time in educating participants and ensuring they understand the contents of the consent forms. In a study conducted on the clinical trial sites in the United States, transportation costs are a major hindering factor to trial participation. Almost all the study respondents stated that recruitment will be improved with transportation efforts (Allisonet al., 2022).

Parkhurst and Froment reported research aimed to understand the practical and psychological barriers to clinical trial diversity and accessibility. Twenty-nine percent (29%) of the participants reported that the time commitment to a clinical trial was not compatible with their daily life, and 24% reported the potential travel costs as the most common practical barriers for participation (Parkhurst and Froment, 2024). Similar findings as regards time constraints were also reported (Al-Shami et al., 2022).

Participants in our study also reported the lack of awareness about clinical research as a barrier to participation. A study was reported in accordance with our results where participants were evaluated to determine whether their willingness to participate in clinical trials would increase by the provision of brief educational material about clinical trials. The results stated a higher likelihood of enrolment in a study after reading the educational material (Allison et al., 2022). Additionally, Participants reported that if they are adequately informed about clinical trial specifics like the potential promise of treatment, its benefits, and toxicity, they will be more willing to participate in the trial (Wong et al., 2020).

Lastly, participants in our study reported that not wanting to change treatment was not a reason for not participating. Although one individual who chose this as a reason stated, “I did not see anything wrong with my body, so why should I change my treatment by participating in this study?.” This response reinforces our understanding that the primary motivation for most people to join clinical trials is to benefit themselves and improve their treatment, as previously reported.

In summary, this study reveals valuable insights into the participants’ attitudes and perceptions of clinical trial participation. The findings have implications for how researchers design clinical trials, especially in settings that lack awareness of clinical research, like Nigeria. The study can guide the development of outreach and recruitment approaches, as well as enhance the understanding of factors that influence participation in clinical trials.

As a recommendation, HIV- related clinical trials or other similar health conditions should be designed in a manner whereby the trial procedures align with the participant’s hospital visits. This will help in patient retention. In addition, researchers should allow patients to assist them in designing their clinical trial protocols to overcome challenges such as patient recruitment and retention. This will reduce cases of lost to follow and withdrawals of participants in clinical research. Additionally, more time and innovative strategies should be employed to explain the content of the informed consent form. These could be achieved by using short films conducted in different local languages, which can be viewed from the mobile phones of study participants. This could improve their understanding of the consent form and may therefore improve participation and retention in clinical trials. Other factors include improved infrastructure support, such as increased research personnel support and giving extra time to explain a clinical trial to potential participants (Wong et al., 2020).

### 4.1 Strengths and limitations of the study

Most studies that reported on the attitudes and perceptions of participants to research participation were conducted on the public (AL-Tannir et al., 2016; Tohid et al., 2017). Our study was conducted on participants in a clinical trial setting. This gives more credence to our study results. Limitations of the study must be highlighted to be alleviated in future research. This includes other factors that could affect the participation or refusal to participate in clinical research that were not addressed in our study. Future studies should include more diverse factors as motivators and/or barriers to participation reported in the literature. Furthermore, our study was conducted on PLHIV. Their perceptions and attitudes to participation in a clinical trial may be reflective of factors uniquely associated with their HIV status. More studies on other health conditions that are endemic in Nigeria should be conducted. Lastly, the views reported in our study were from a small sample of participants in our moringa clinical trial. The results may not be generalized to other trial settings.

## 5 Conclusion

Patient participation in clinical trials that are well-designed and well-conducted forms the essence of all clinical research. Nigeria offers a naïve patient pool with a varied range of disease conditions. Improving awareness will improve patient participation and retention in clinical trials, which will invariably increase access to the newest available treatments.

## Data Availability

Data cannot be shared publicly due to the ethical restrictions regarding patient confidentiality imposed by the ethics committee of Aminu Kano Teaching Hospital, Kano state, Nigeria and University of Kwazulu- Natal, Durban, South Africa. Requests to access the datasets should be directed to Biomedical Research Ethics Committee of University of Kwazulu-Natal, Durban South Africa. Westville Campus, Govan Mbeki Building. Postal address: Private Bag x54001, Durban 4000. Tel: +27(0) 31 260 2486, Facsimille: +27(0) 31 260 4609. Email: brec@ukzn.ac.za.
