# Supplementary material for: Perceptions and attitudes toward clinical trial participation: a study on Moringa oleifera Lam. supplementation in adult HIV patients in Kano State, Nigeria
Source: Front Pharmacol. 2025 Oct 31;16:1676393. doi: 10.3389/fphar.2025.1676393 (PMC12615447; doi:10.3389/fphar.2025.1676393)
Supplement: Supplementary file 1 [file Table1.docx]

**Group A**

**Those who have participated fully in the Moringa clinical trial (visit 1 to 5,6 or 7)**

**Title: Perceptions and Attitudes to clinical trial participation: A study on Moringa supplementation on adult HIV patients in Kano State, Nigeria**

**MC NO**: Date:

**Sociodemographic characteristics of participants**

| **Gender** |
| --- |
| Males |
| Female |
| **Age (years)** |
| < 20 |
| 20-29 |
| 30-39 |
| 40-49 |
| 50-60 |
| **Educational Level** |
| Primary |
| Secondary |
| Tertiary |
| Quranic |
| None |
| **Occupation** |
| Entrepreneur |
| Trader |
| Civil Servant |
| Artisan |
| Unemployed |

- Have you ever been approached or invited to participate in a clinical trial study in the past? If yes, then
- What disease?.....................

**Section 1: Participants will be asked to rate their reasons for participation in clinical research on a scale of 1–3, where**:

1. most important
2. second most important
3. third most important

**The different reasons for participation in clinical research are:**

1. I was curious
2. To help myself
3. Thought it might improve my access to healthcare/ improve my treatment
4. Because I was asked by the doctor
5. I felt I had to
6. I did not want to say no
7. To help others
8. I do not know
9. For the money

**Section 2: Furthermore, group A respondents will be asked the following agreement and disagreement statements related to participation in clinical research.**

1. I gained something positive from participating.
2. Knowing what I know now, I would participate in clinical research if given the opportunity.
3. The research raised emotional issues for me that I had not expected.
4. I gained insight about my experiences through research participation.
5. The research made me think about things I didn't want to think about.
6. I found the questions too personal.
7. I found participating in the clinical research personally meaningful.
8. I believe the clinical research results will be useful to others.
9. I trusted that my replies would be kept private.
10. I experienced intense emotions during the research session and/or parts of the study.
11. I think clinical research is for a good cause.
12. I was treated with respect and dignity when I participated in clinical research.
13. I found participating in clinical research beneficial to me.
14. I was glad to be asked to participate.
15. I like the idea that I contributed to science.
16. I was emotional during the research session.
17. I felt I could stop participating at any time.
18. I found participating boring.
19. The study procedures took too long.
20. Participating in clinical research was inconvenient for me.
21. Participation was a choice I freely made
22. Had I known in advance what participating would be like I still would have agreed to participate.
23. I understood the consent form.
