# Supplementary figures and images for: Perceptions and attitudes toward clinical trial participation: a study on Moringa oleifera Lam. supplementation in adult HIV patients in Kano State, Nigeria
Source: Front Pharmacol. 2025 Oct 31;16:1676393. doi: 10.3389/fphar.2025.1676393 (PMC12615447; doi:10.3389/fphar.2025.1676393)

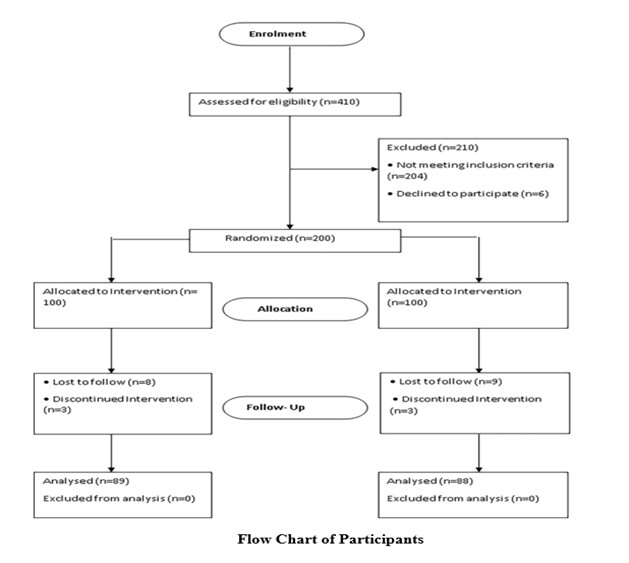

Supplement: Supplementary file 2 [file Image1.jpeg]
