# Supplementary material for: Perceptions and attitudes toward clinical trial participation: a study on Moringa oleifera Lam. supplementation in adult HIV patients in Kano State, Nigeria
Source: Front Pharmacol. 2025 Oct 31;16:1676393. doi: 10.3389/fphar.2025.1676393 (PMC12615447; doi:10.3389/fphar.2025.1676393)
Supplement: Supplementary file 3 [file Table2.docx]

**Group B**

**Those who had declined participation or dropped out from the Moringa clinical trial**

**Title: Perceptions and Attitudes to clinical trial participation: A study on Moringa supplementation on adult HIV patients in Kano State, Nigeria**

**MC NO**: Date:

**Sociodemographic characteristics of participants**

| **Gender** |
| --- |
| Males |
| Female |
| **Age (years)** |
| < 20 |
| 20-29 |
| 30-39 |
| 40-49 |
| 50-60 |
| **Educational Level** |
| Primary |
| Secondary |
| Tertiary |
| Quranic |
| None |
| **Occupation** |
| Entrepreneur |
| Trader |
| Civil Servant |
| Artisan |
| Unemployed |

- Have you ever been approached or invited to participate in a clinical trial study in the past? If yes, then
- What disease?.....................

**Section 1: Participants will be asked the following reasons for their refusal to participate in the clinical trial with a ‘YES’ or ‘NO’.**

1. I don't want to change treatment
2. I am too ill
3. Possible side-effects of tests
4. I don't want to be a guineapig
5. I am too old
6. I don't have enough time
7. Fear
8. I don't like hospitals
9. Lack of awareness about clinical research
10. Family objected
11. Lack of interest in research
12. I don’t have enough money to be coming to the hospital for the research
